# The role of sarcopenia in determining the risk of adverse events in atrial fibrillation: advancing the need for a geriatric approach

**DOI:** 10.1093/europace/euaf310

**Published:** 2025-12-02

**Authors:** Marco Proietti, Anna Ronca, Giuseppe Boriani

**Affiliations:** Department of Clinical Sciences and Community Health, University of Milan, Via Commend 19, Milan 20122, Italy; Division of Cardiogeriatric Subacute Care, IRCCS Istituti Clinici Scientifici Maugeri, Via Camaldoli 64, Milan 20138, Italy; Department of Clinical Sciences and Community Health, University of Milan, Via Commend 19, Milan 20122, Italy; Geriatric Medicine Specialty Training School, University of Milan, Milan, Italy; Cardiology Division, Department of Biomedical, Metabolic and Neural Sciences, University of Modena and Reggio Emilia, Policlinico di Modena, Modena, Italy

## Abstract

**Graphical Abstract:**
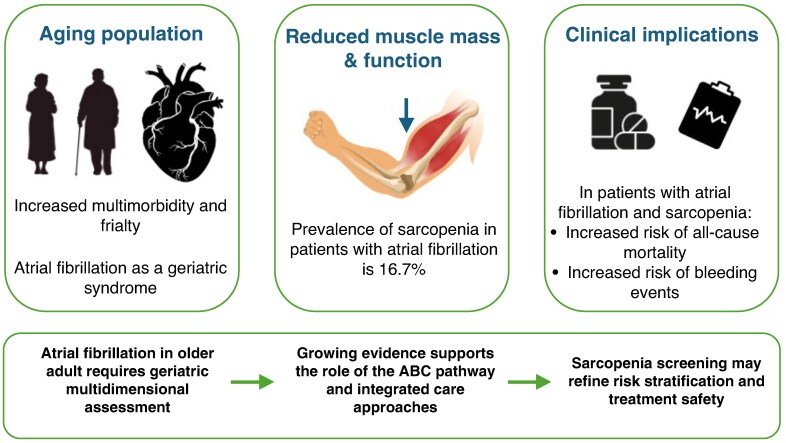
ABC, Atrial fibrillation Better Care.

ABC, Atrial fibrillation Better Care.

**This editorial refers to ‘Sarcopenia in atrial fibrillation: a risk factor for adverse outcomes in a UK biobank study’ by H.J. Kim *et al*., https://doi.org/10.1093/europace/euaf286.**

The on-going global ageing of the population has introduced a spectrum of complex challenges for contemporary cardiovascular medicine. Older adults frequently present with a combination of multimorbidity, physical frailty, muscle loss, cognitive impairment, incontinence, and malnutrition, all of which are associated with poorer clinical outcomes, diminished quality of life, and heightened healthcare utilization.^1^ In parallel, atrial fibrillation (AF) has become increasingly prevalent with advancing age, reaching its highest incidence in the oldest segments of the population.^2^

These trends underscore the importance of integrating age-specific strategies for the prevention, diagnosis, and management of AF. In fact, AF rarely occurs in isolation; it frequently coexists with multiple chronic diseases and geriatric syndromes, altering treatment priorities and complicating decision-making. Recent research highlights that AF incidence is influenced not only by traditional cardiovascular risk factors but also by age-related conditions, which contribute to polypharmacy, inappropriate prescribing, underutilization of oral anticoagulants, and heightened risk of adverse outcomes.^3,4^ As a result, older adults with AF have diminished independence in activities of daily living and are disproportionately affected by frailty.^5^ Estimates suggest that frailty may be present in up to 40% of patients with AF,^5^ and population-based studies report a strikingly high mortality rate—approximately 20–25%—in the first year after a new AF diagnosis in older adults.^4^

Geriatric syndromes, by definition, arise from multisystem impairments and cannot be neatly explained by a single pathophysiological process. Instead, they represent interconnected vulnerabilities that lead to unpredictable trajectories and disproportionate clinical consequences. AF fulfils several characteristics of a geriatric syndrome: it is highly prevalent in older adults, strongly associated with multimorbidity and frailty, linked to cognitive and functional decline, and deeply impactful on quality of life. Reconceptualising AF as a geriatric syndrome strengthens the argument for comprehensive, multidimensional care models tailored to the needs of older patients.^6^ Among the various factors possibly influencing the onset and progression of AF, sarcopenia has gained particular interest. Defined as a progressive skeletal muscle disorder marked by reduced muscle mass and function, sarcopenia is associated with fractures, osteoporosis, increased hospitalization, and poor quality of life.^7^

In this context, the study by Hong-Ju Kim *et al.* adds valuable insight by examining the relationship between sarcopenia and clinical outcomes in patients with AF, using longitudinal data from the UK Biobank.^8^ In this large retrospective cohort, sarcopenia was identified in 16.7% of patients with AF. Those with sarcopenia exhibited a higher comorbidity burden and experienced significantly more adverse events. The primary composite outcome—which encompassed all-cause mortality, stroke, systemic embolism, major bleeding, clinically relevant non-major bleeding, and hospitalization—occurred significantly more frequently in patients with both AF and sarcopenia compared to those with AF alone (43.9 vs. 35.1 per 1000 person-years, adjusted hazard ratio (HR) 1.30; 95% CI: 1.15–1.46). Notably, bleeding events were the strongest driver of risk (adjusted HR, 1.34; 95% CI: 1.10–1.65). Although rates of stroke, systemic embolism, and heart failure hospitalization were also numerically higher, these associations did not reach statistical significance. Several limitations warrant consideration. As with all analyses based on administrative and volunteer cohort data, residual confounding persists, and the relatively low prevalence of sarcopenia reflects a healthy-participant selection bias in the UK Biobank. Additionally, the study cohort was enrolled before widespread adoption of direct oral anticoagulants, limiting the generalizability of bleeding risk estimates to current clinical practice.

Despite these caveats, the findings are consistent with growing evidence linking sarcopenia, AF, and adverse clinical outcomes. Observational studies suggest that sarcopenia increases the risk of developing AF in older adults (*Table 1*), potentially in a synergistic manner with other age-related conditions.

**Table 1:** Summary of the studies that evidence a link between atrial fibrillation and sarcopenia

Study, year	Geographic location	Study characteristics	Population characteristics	Results
Requena Calleja *et al*., 2019	Spain	Prospective multicentre cohort study	596 Hospitalized patients older than 75 years with non-valvular atrial fibrillation from internal medicine departments (mean age: 84.9 ± 5.2)	Sarcopenia (49.5%), frailty (51.2%), and cognitive impairment (42.1%) are very common in older patients with non-valvular atrial fibrillation; sarcopenia was associated with increased mortality (HR: 1.775; 95% CI: 1.270–2.481)
Xia *et al*., 2021	China	Cross-sectional analysis	2432 Community-dwelling adults from the Shanghai Changfeng Study (mean age: 62.2 ± 8.4)	In middle-aged and elderly adults without clinical heart failure, sarcopenia is associated with AF (OR: 5.67 [1.67–19.24], *P* = 0.005)
Tang *et al*., 2024	United Kingdom	Prospective cohort study	384 433 community-dwelling individuals aged 40–69, from the UK Biobank project (median age 58 years, IQR: 50–63)	Sarcopenia increases the risk of developing AF (HR: 1.08, 95% CI 1.42–2.45); handgrip strength (HR: 1.09, 95% CI 1.04–1.15) and low muscle mass (HR: 1.21, 95% CI: 1.09–1.33) associated with increased AF risk
Shim *et al*., 2024	Korea	Cross-sectional analysis	2225 Participants from the Korean Frailty and Aging Cohort Study (mean age: 76.0 ± 3.9)	Sarcopenia was associated with AF in the cross-sectional analysis (OR: 2.127, 95% CI: 1.240–3.648, *P* = 0.006); among sarcopenia components, low physical performance is significantly associated with AF (OR: 1.872, 95% CI: 1.123–3.120, *P* = 0.016)
Liu *et al*., 2025	China	Prospective cohort study	8060 Non-diabetic community-dwelling older individuals (mean age: 68.4 ± 6.12)	In the prospective analysis, AF risk is increased in patients with sarcopenia (HR: 1.762, 95% CI: 1.528–2.032)
Yu *et al*., 2025	China	Prospective cohort study	4321 Non-diabetic community-dwelling older adults (mean age: 68.5 ± 6.18)	Sarcopenia and obesity increased AF risk acting in a synergistic way (HR: 2.029, 95% CI: 1.639–2.512)

AF, atrial fibrillation; UK, United Kingdom; HR, hazard ratio; CI, confidence interval; OR, odds ratio; IQR, interquartile range.

The biological mechanisms driving sarcopenia are multifaceted and may include epigenetic alterations, impaired proteostasis, and depletion of muscle stem-cell reserves.^7^ While standardized pharmacological strategies for treating sarcopenia remain elusive, interventions such as resistance training and adequate protein intake have demonstrated meaningful improvements in muscle mass, strength, and physical performance.^9^ Physical activity, in particular, reduces risks commonly associated with sarcopenia, including falls, disability, and hospitalization.^7^ These findings raise the question of whether screening for sarcopenia—now feasible through simple, validated tools^7^—should be incorporated into routine AF evaluation.

The complexity of AF in this population requires a multidimensional assessment, which includes evaluation of comorbidities, geriatric syndromes, polypharmacy, and functional and cognitive status, as emphasized in recent reviews and in the *European Society of Cardiology 2024 Guidelines*.^10^ Such an approach supports individualized management and optimizes therapeutic decisions, particularly regarding anticoagulation and rhythm control. As a strategy to streamline the application and implementation of a holistic care approach, the ABC (Atrial Fibrillation Better Care) pathway, based on the three pillars of ***A***void stroke by anticoagulation prescription, ***B***etter symptoms management with rate and rhythm control strategies, and ***C***omorbidities, risk factors and lifestyle habits optimization, has been developed. This model provided a foundation for the construction of subsequent interactions, emphasizing the importance of multidisciplinary collaboration, patient empowerment, and the utilization of digital tools for self-management. A clinical management strategy based on the ABC pathway has been shown to effectively reduce the risk of various clinical outcomes, as demonstrated in randomized clinical trials^11,12^ and further corroborated by retrospective observational data.^13^ Within this framework, incorporating sarcopenia screening could refine risk stratification and facilitate safer, more tailored treatment decisions.^8^

Large-scale initiatives such as the *Atrial Fibrillation Integrated approach in Frail, Multimorbid and Polymedicated Older People* (AFFIRMO) programme embody this evolution in care philosophy. Since its beginning, AFFIRMO has aimed to evaluate the impact of multimorbidity in AF and to determine whether patient-centred, stratified care—built upon the ABC pathway and enhanced by comprehensive geriatric assessment—can reduce major adverse outcomes in older European patients.^14^ The programme’s central hypothesis is that integrated, multidisciplinary care is superior to traditional disease-oriented management, particularly in populations with the highest burden of frailty, multimorbidity, and functional impairment.^14^

AFFIRMO marks a crucial step towards integrating geriatric principles into cardiovascular care and may offer definitive evidence, upon the completion of the currently ongoing RCT, to support the routine adoption of such approaches, alongside other studies evaluating comprehensive comorbidities management in AF patients.^15^

A systematic, structured approach should be promoted and prioritized to ensure the thorough, organized management of patients with AF. Sarcopenia, increasingly recognized as both a marker and mediator of vulnerability, may play an important role in shaping clinical outcomes in older adults with AF. Future research should clarify whether sarcopenia-targeted interventions can modify the trajectory of AF or reduce adverse events, particularly bleeding risk.

As we await the results of on-going trials, including AFFIRMO, the evidence to date supports a shift towards personalized, multidimensional, and integrated care strategies that bridge cardiology and geriatrics. The convergence of AF, frailty, multimorbidity, and sarcopenia underscores the need to move beyond narrow disease-specific frameworks. Doing so will be essential to improving the lives of the rapidly growing population of older adults living with AF.

## Data Availability

No new data were generated or analysed in support of this research.
